# Mathematics Disability vs. Learning Disability: A 360 Degree Analysis

**DOI:** 10.3389/fpsyg.2021.725694

**Published:** 2021-09-24

**Authors:** Roderick Ian Nicolson, Angela Jocelyn Fawcett

**Affiliations:** ^1^ Department of Psychology, Edge Hill University, Ormskirk, United Kingdom; ^2^ Department of Psychology, Swansea University, Swansea, United Kingdom

**Keywords:** mathematics disability, reading disability, learning and skill acquisition, affective, developmental

## Abstract

A fundamental issue for research in mathematics disability (MD) and reading disability (RD) is: If these disabilities are clearly distinct, why is there so high a level of comorbidity, together with the converse; if these disabilities are so similar, why are there clear differences in underlying causes and aetiology? In order to address this puzzle, we introduce the “360 degree analysis” (360DA) framework and apply it to the overlap between RD and MD. The 360DA process starts by analyzing the issue from four perspectives: theoretical, developmental, affective, and pedagogical. Under 360DA, these analyses are then integrated to provide insights for theory, and for individual assessment and support, together with directions for future progress. The analyses confirm extensive similarities between arithmetic and reading development in terms of rote learning, executive function (EF), and affective trauma, but also major differences in terms of the conceptual needs, the motor coordination needs, and the methods of scaffolding. In terms of theory, commonalities are interpreted naturally in terms of initial general developmental delay followed by domain-independent affective trauma following school failure. Dissociations are interpreted in terms of cerebellar vs. hippocampal learning networks, sequential vs. spatial processing, and language vs. spatial scaffolding, with a further dimension of the need for accurate fixation for reading. The framework has significant theoretical and applied implications.

## Introduction

This article contributes to the special issue on the links between mathematics disability (MD) and reading disability (RD). It builds upon our earlier analysis (Nicolson and Fawcett, 2007), in which we highlighted the need for a re-convergence of research and practice in learning disabilities. In the article, we highlighted the decades of increasing differentiation after the 1970s, with each disability developing its own paradigm, from theory to practice to support structures, whereas the high comorbidity between the disorders suggested that there might well be common factors. At the theoretical level, we built on the long-established distinction between procedural and declarative memory (Anderson, 1982; Squire et al., 1993) to introduce the distinction between declarative learning (scaffolded by the declarative networks, with key structure: the hippocampus) and procedural learning (scaffolded by the procedural system, with key structures: the cerebellum and the basal ganglia). In the intervening years, the existence of these networks has been fully established (Bostan et al., 2013; Buckner and Krienen, 2013), and the strength and breadth of the comorbidities have also been fully established. Nonetheless, these insights do not appear to have led either to greater convergence of theory or to significant changes in support, as evidenced by the need for special issues such as this.

In this article, we aim to stimulate fruitful convergence between the different paradigms for learning disabilities by advocating a doubly integrated approach, which attempts to find commonalities between the learning disabilities and also to provide a broad framework that integrates the diverse theoretical and applied approaches to their analysis and treatment. The 360 degree analysis (360DA) process provides all-round analysis from all angles, by analogy with the best-practice approaches developed for appraisals in business. Our focus here is on MD vs. RD, but the approach is of general applicability. Our own research specialism is in RD, and so we have limited our detailed analyses to RD, in the hope that MD specialists will feel empowered to undertake similar analyses themselves.

Let us begin by laying out the issues regarding RD and MD established *via* the rightly influential study of Willcutt et al. (2013, p. 500) of twins within the Colorado Learning Disabilities Research Center program. The authors concluded that “*Groups with RD only, MD only, and RD+MD were significantly impaired vs. the control group on nearly all measures, and the group with RD+MD was more impaired than the groups with MD and RD alone on measures of internalizing psychopathology, academic functioning, and seven of 10 neuropsychological constructs… deficits in reading and math were associated with shared weaknesses in working memory, processing speed, and verbal comprehension. In contrast, reading difficulties were uniquely associated with weaknesses in phoneme awareness and naming speed, and math deficits were uniquely associated with weaknesses in set shifting*.” It is important to note in the light of our discussion below that the researchers declined to use a discrepancy criterion and do not report gender analyses.

A key finding with respect to this special edition is that the numbers diagnosed with RD only, MD only, and RD+MD were 241 (39%), 183 (30%), and 188 (31%), respectively, highlighting the high comorbidity between the disorders. As Gilger and Kaplan (2001) argue, “*in developmental disorders comorbidity is the rule not the exception*,” as ably discussed by contributors to this issue.

In terms of incidence, percentages depend critically on the criteria used – one is essentially placing an arbitrary cutoff on the normal distribution – and whether or not a strict inclusionary criterion is applied. The traditional specific learning disability definition was: “a disorder in children who, despite conventional classroom experience, fail to attain the language skills of reading, writing, and spelling commensurate with their intellectual abilities” (from the definition by the World Federation of Neurology, 1968, p. 26). The definition is an exclusionary one, serving to exclude children of low overall intelligence from being diagnosed with dyslexia. If exclusionary criteria are omitted, as has been practiced in the United States (Stuebing et al., 2002; Fletcher et al., 2004), thereby including any child struggling with reading, or math, or attention, the incidence at least trebles, comorbidity balloons, and theoretical explanations become hopelessly confounded (Nicolson, 1996; Stein, 2018).

Traditional estimates for the male/female ratio for RD as specific learning disability were around 4:1 (Miles et al., 1998), though inclusionary estimates suggest ratios closer to parity (Shaywitz, 1998). By contrast, more girls than boys suffer from MD; especially, if math anxiety is taken into account (Goetz et al., 2013), though again ratios approach parity with inclusionary criteria (Devine et al., 2013). A recent study in Germany (Moll et al., 2014) investigated reading disorder, and spelling disorder (SD) and arithmetic disorder (AD) in 1633 grade 3 or 4 children. They established high comorbidity rates between all three disorders, and (perhaps, surprisingly) that SD co-occurred more frequently with AD than with reading disorder. Gender ratios varied somewhat with the deficit criterion used, but for comorbid reading+spelling disorder (equivalent to RD) there were 60% male, whereas for AD (equivalent to MD) there were around 35% male. It is inevitable that the ratios do approach parity as the inclusionary criteria are relaxed owing to the mixing of the preponderance of boys with RD and girls with MD. We will use the gender dissociation between specific RD and specific MD as a theoretical scalpel to tease apart the two learning disabilities.

We complement the other contributions by picking out three fundamental issues for comparison of reading disability and math disability: Why are there overlaps, why are there differences, and what are the implications for subsequent progress? We advocate the importance of the 360DA process combining theoretical, cognitive, affective, and educational perspectives, arguing that these may all be integrated within a task analysis perspective.

## A 360 Degree Analysis

We propose the 360DA as a means of combining the multiple different perspectives provided by the increasing depth and narrowness of scope provided by increasing specialization of methodology (Nicolson et al., 2021). It is intended to allow integration of a number of perspectives – individuals as well as groups, strengths as well as weaknesses, future opportunities as well as past and present performance, emotion as well as cognition, the situation as well as the person, and 360 degree assessment, theory, support, and involvement. In this paper, we first consider four major perspectives for developmental disorders: theory, development, affect, and pedagogy. Following these analyses, we then attempt to provide an integration in terms of answers to key questions, including the theoretical and pedagogical implications.

### The Theoretical Perspective: History

In terms of underlying theory, it is valuable (Morton and Frith, 1995) to distinguish different levels of explanation – the behavioral level (symptoms), the cognitive (mental function) level, and the brain (structure or function) level, and now the genetic level. Nicolson and Fawcett (2007) advocate the introduction of a neural network-level intermediate between the cognitive level and the brain level.

For RD, ongoing behavioral symptoms are reading dysfluency and developmental delay in phonological processing, with the initial symptoms being delay in phonemic awareness. At the cognitive level, there is a variety of theories advanced to explain subsets of the underlying symptoms. The leading theory remains the phonological deficit theory (Stanovich, 1988; Vellutino et al., 2004). Alternative prominent frameworks include the magnocellular deficit theory (Stein, 2001), which is at the brain level, and the automatization deficit theory (Nicolson and Fawcett, 1990) at the cognitive level which was followed up by the cerebellar deficit theory (Nicolson et al., 2001) at the brain level and the procedural deficit theory (Nicolson and Fawcett, 2007) at the neural networks level. There are also theories at the visual attention level faculty, for example, Facoetti et al. (2003), and current theorists have incorporated within the phonological deficit framework the additional elements of the double-deficit theory, namely working memory and speed of processing (Vellutino et al., 2004). The original double-deficit hypothesis (Wolf and Bowers, 1999) gained force from the demonstration that remediation was more difficult for children with a double deficit – both in phonological processing and in speed of processing – than for those with just a single deficit.

For MD, the symptoms are arithmetic dysfluency and developmental delay in acquiring the mathematical milestones together with persistent failure in acquiring number bonds. Two broad hypotheses – numerosity deficit and symbol-magnitude mapping deficit – advocate a domain-specific disorder, whereas the executive function (EF) hypothesis posits domain general issues. The numerosity deficit hypothesis proposed a core deficit in processing quantity (Butterworth et al., 2011) and number sense (Dehaene et al., 2004; Decarli et al., 2020). The symbol-magnitude mapping deficit hypothesis proposed a specific weakness in automatically mapping symbols to their internal magnitude representations (Rubinsten and Henik, 2006; Rousselle and Noel, 2007). The domain general EF deficit highlighted problems to working memory (Rotzer et al., 2009; Toll et al., 2011) and attention (Ashkenazi et al., 2009), rather than as a specific deficit in number processing.

We return to theoretical frameworks within the integration section.

### The Developmental Perspective

One of the most potent critiques (Frith, 2001; Goswami, 2003) of the naive application of information processing measures such as working memory, processing speed, executive function, and attention to developmental disabilities is that this approach does not naturally take account of developmental processes within individuals as pioneered by Inhelder and Piaget (1958), Bruner (1960), Flavell (1977), and Vygotsky (1986). In particular, of course, a single cross-sectional analysis of differences in attainment is unable to establish the underlying causes and can only be considered a description rather than explanation.

In response to such critiques, there have been systematic attempts to undertake longitudinal, multi-year analyses, whereby a cohort of children at risk of dyslexia are followed through from birth to say 10years of age. Two such studies for dyslexia, the Jyyvyskala study (Lyytinen et al., 2004) in Finland and (Blomert and Willems, 2010; van der Leij, 2013) in the Netherlands, provided a wealth of information, but unfortunately without clear theoretical implications.

A recent smaller-scale longitudinal study, directed by Snowling et al. (2021), provides a valuable illustration of the strengths (and pitfalls) of a non-developmental longitudinal analysis. The study followed three groups: language disorder (LaD), risk of RD and typical development (TD) from 4years to 8years, using sets of age-appropriate tests annually for tests 1–5 (T1–T5). An initial study (Gooch et al., 2014) identified that both language skill and motor skill contributed unique variance from T1 to T2. Of particular interest here, the team were able to investigate MD as well as RD, concluding (Snowling et al., 2021) that “*Poor language was associated with each disorder and appears to be a cognitive risk factor for RD, MD, and RD&MD*” (abstract). The basket of tests, though large, was not designed to test between theories and (with the exception of motor skill) used tests that would be expected to reveal difficulties given the expanded phonological deficit framework and, therefore, did not include measures of speech rate, sensory processing ability, eye movement control, or internalized speech, thereby limiting the scope for theoretical progress.

The authors did not report individual analyses, as would be undertaken at school for an Individual Education Plan, and so it was not possible to analyze the individual responses to interventions administered, although, presumably the majority of the LaD and Risk of RD participants would have been receiving additional support by the school. Furthermore, as highlighted by all the influential developmental psychologists from Inhelder and Piaget (1958) and Vygotsky (1986) to Frith (1986) and Goswami and Bryant (1990), development of skills occurs in a series of stages, with fluency in some sub-skills being the prerequisite for acquisition of more complex skills, whether those skills be motor, cognitive, or executive function.

### The Affective Perspective

It is now established that dyslexic individuals of any age suffer more stress and “internalising disorders” than their typically achieving peers (Livingston et al., 2018; Francis et al., 2019; Haft et al., 2019). Surprisingly, this insight has not been explicitly integrated into theoretical or educational approaches to dyslexia. By contrast, “math anxiety” has been extensively studied. It is present even at the beginning of formal schooling (Maloney, 2012). Furthermore, the anxiety was triggered by anticipation rather than performance of mathematics tests (Lyons and Beilock, 2012), supported by brain imaging studies that indicated that the cognitive information-processing deficits arising from mathematics anxiety can be traced to brain regions and circuits that have been consistently implicated in specific phobias and generalized anxiety disorders in adults (Young et al., 2012).

In terms of situation-specific stress, the stressor leads to hypothalamic–pituitary–adrenal (HPA) activity causing cortisol to be released, diverting blood from the brain to the muscles for the “fight, flight, or freeze” response. Even mild stressors actually affect the brain circuits involved in learning. Basically, stress shifts processing from the declarative system to the action-based procedural system – the fight or flight – as one might expect in order to escape from that situation (Schwabe and Wolf, 2013).

After prolonged stress, changes in plasticity take place, primarily in the neural circuits involving the hippocampus and amygdala (McEwen, 2012). This impairs hippocampal function, which can have two effects: to impair hippocampal involvement in episodic, declarative, contextual, and spatial memory and to impair hippocampal regulation of hormonal regulation *via*, particularly the termination of the stress response, leading to elevated HPA activity and further exacerbating the actions of adrenal steroids in the long-term effects of repeated and chronic stress exposure.

Unfortunately, even a single failure in the presence of classmates leads to a feeling of shame. Like stress, shame leads to greater expression of cortisol (Gruenewald et al., 2004), even in 4-year-old children (Lewis and Ramsay, 2002). Children with learning weaknesses in a particular domain are subjected to continual stressful and shame-inducing experiences that singly and in combination may lead to their escalation into full-blown learning disorders in that domain, and these toxic experiences may lead to generalization to other domains of schoolwork.

### The Pedagogical Perspective

This perspective includes two aspects: first the learning processes involved and then task analysis for the skills in question.

#### The “Learning Process” Approach

A particularly surprising omission in research on the learning disorders, highlighted initially by Nicolson and Fawcett (1990), is the failure of almost all research – cross-sectional or longitudinal – to analyze the processes of learning itself, to distinguish between “learning product” (attainment) and “learning process” (registration, execution, consolidation, automatization, for example). In their “Dyslexic Automatisation Deficit” theory, they established that their dyslexic participants showed incomplete task automaticity (as revealed by deficits under dual task conditions) and attributed this to dysfunction in one of the many “learning” processes involved in skill automatization. In subsequent work, they established differences in the long-term acquisition of skill on a simple reaction time game (Nicolson and Fawcett, 2000), on lack of cerebellar activation on acquiring a simple motor sequence (Nicolson et al., 1999), on eye blink conditioning (Nicolson et al., 2002), on adapting to prisms (Brookes et al., 2007) and various other forms of long-term procedural learning (Nicolson et al., 2010).^1^ These studies led to their cerebellar deficit theory (Nicolson et al., 2001), their specific procedural deficit theory (Nicolson and Fawcett, 2007), and their recent Delayed Neural Commitment theory (Nicolson and Fawcett, 2019), in which they provided a plasticity-based framework for understanding the underlying learning differences. The key point, however, is that any theoretical or empirical approach that does not look at the fine grain of the processes of skill acquisition (rather than the products) is not able to provide a rationale for change in educational processes for any given child.

#### The Task Analysis Perspective

One of the most programmatic and influential analyses of learning and instruction was provided by Gagne (1965), and although, now dated it provides a valuable resource for anyone wishing to explore methods of instruction in greater depth. In brief, Gagne (1965) distinguished five key capabilities, namely information, intellectual skills, cognitive strategies, motor skills, and attitude. Theory of instruction of Gagne (1965) suggests that the overall method for training any skill is first to do a thorough task analysis, determining what the sub-skills involved are, and what their pre-requisites are and so on, thus identifying a hierarchical tree of sub-skills that make up the skill. For each sub-skill, it is necessary to determine which of the five types of capability it is, and then to devise a method for instruction on that sub-skill.

##### Early Mathematics

It may help to start with an informal task analysis for the early stages in mathematics development. One of the earliest stages involves the realization that objects are discrete, and then, grasping the idea that similar objects can be combined together with the numbers 1, 2, and 3 corresponding to groups of bigger size. This conceptual revolution is often scaffolded by linking the number to a sequence of actions – one clap, two claps; one step, two steps; one finger, two fingers, and so on. These concepts can then be applied to a range of objects and actions, and reinforced by repetition. The graphemes to represent one and two on paper come later, but there are only 10 including zero.

Conceptually after the fingers, the discrete number line is a key concept, with implicitly the idea of equal intervals between each unit in the sequence. Strips of paper or even a ruler can be used to help with this concept. Place order is one of the most challenging early concepts, typically requiring considerable practice – laying out the blocks in the spatial block fashion so that, for instance, 12 is represented as 10+2. Dienes’ blocks provide a common scaffolding involving the concept of area. The number facts 8+5 and the times tables are generally learned by rote and may be seen as a form of association learning.

Even this simplistic analysis allows us to identify a number of different component knowledge and skill aspects to mathematics. We have the conceptual level: the very idea of number; the number fact level; the place order convention; and then the operator for addition. Of course the operator plus is the simplest one, often scaffolded by using blocks to manually represent the process. The visual grapheme “+” is of course an item to learn. We are grateful to a referee for pointing out that a key conceptual advance needed is that of unitizing, wherein the two addends somehow become equivalent to the one outcome. The next operation typically is subtraction, which is generally shown as the inverse operation for addition, and addition and subtraction can relatively easily be facilitated using the fingers for sums going to less than 10. Multiplication is a more complex mental conceptual idea, almost certainly scaffolded by the concept of repeated addition – so three sixes are six and six and six. This is often most easily shown in the way of a two-dimensional spatial representation, which can then be counted. Division is the most complex operation, often scaffolded by the concept of dividing a cake into parts, but is made particularly complex by having to unlearn the concept of discrete units 1, 2, and 3 and replace this with a more continuous line as one sees, for example, on the ruler.

There is clearly an explicit “skill building” program needed, with first the number concepts, then the addition operator and addition facts, then the subtraction process, operator and facts, then the multiplication process, operator, times tables, and finally the fourth operator, division. Without fluent knowledge of the times tables, non-trivial division sums just cannot be done effectively.

In order to be fluent in early arithmetic, it is therefore important to have the conceptual development necessary, the understanding of the operators and how they work and how they are different, to have built up a series of number bonds or factual knowledge base. For fluency, it is necessary to have internalized all of these facts and processes so that they are accessed automatically, leaving full working memory to complete any sums. Key scaffolders include one’s fingers and actions, external objects, the number line, and spatial arrays to represent area.

It is also important to highlight the EF skills required for early arithmetic learning. There is the essential requirement of what one might call “math readiness.” This is strongly related to the concept of “reading readiness” (Petty, 1939), which was a major explanatory construct throughout the mid-twentieth century, but was downplayed in mainstream reading instruction (despite its emphasis in Reading Recovery) until re-emerging in the twenty-first century (Duncan et al., 2007). A key development is the recent literature on the development of executive function from 3 to 6years, and in particular the role of executive functions in “school readiness” (Fitzpatrick et al., 2014). An early review (Blair, 2002) highlighted the importance not only of the “cool EF” capabilities described above but also the emotional and social EF (“hot EF”) requirements for school readiness.

Both reading readiness and classroom readiness are needed to benefit from classroom teaching of reading. Reading readiness includes established skills such as phonological awareness and letter knowledge, together with appropriate eye fixation control and knowledge about print. Classroom readiness includes both the cool EFs of working memory, response inhibition and task maintenance, and the hot EFs of emotional control, anger control, and aggression control.

##### Learning to Read

Acquisition of reading does overlap in many aspects, but is fundamentally different in several key dimensions. Unlike mathematics, the fundamental underpinning of reading – language comprehension – is already established well before reading instruction takes place. The key conceptual requirement therefore is to attempt to map the print symbols on the printed page onto the established language capabilities.

Print is an abstract, written representation of speech. It omits salient aspects of speech, such as loudness, pitch, emphasis, emotion, and prosody. It introduces gaps between words which are not there in speech, and it provides a “one size fits all” representation for all the many pronunciations of any given word.

Over and above these distortions, it introduces a classification of words into syllables and of syllables into phonemes that is both abstract and arbitrary. The phonemes are then mapped on to letters in a way that is neither consistent nor transparent. Finally, in order to read a word it is necessary to fixate each grapheme on the page in turn, to say the corresponding phoneme, to blend in the next phoneme, and hopefully to map the utterance onto the underlying representation for the target word.

There are, therefore, similarities with math learning: There is a need to learn the “letter facts,” the graphemes and their link to phonemes; there is a clear skill of holding phonemes in working memory, while acquiring the next one to blend with it. Conceptually, however, the major issue is that of analysis – that spoken words can split into syllables (which are meaningless) and that syllables can be split into phonemes – which are even more meaningless (and may be poorly represented in the learner’s expressive speech skills).

Conceptually, there is a need to recognize that the words in a book go across the page from left to right (in English), and the lines go down the page (Clay, 1993), but there is no scaffolding provided by spatial characteristics of the world (the number line, the spatial area, and the fraction of pie). By contrast with mathematics, there is an overwhelming need to be able to fixate accurately on a single grapheme for the time needed to recognize it, to move fixation accurately to the next letter, recognize that, register both letters in working memory, move to the end of the word letter by letter, then assemble the component phonemes into the corresponding sound, and recognize the sound as a word, all the time retaining the location of the target word in visual working memory, so that the following word may then be fixated.

**Figure 1:**
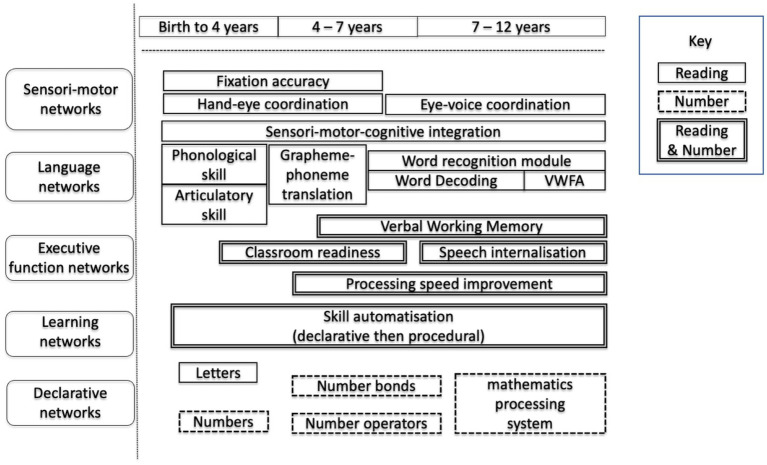
The development of reading skill and mathematics skill.

Figure 1, adapted from our contribution to Marien et al. (2014) through the inclusion of the development of mathematics skills, highlights the interplay between the procedural and executive skills of speed, language, fixation, and phonics in early reading development (single line) compared with the much stronger contribution of declarative skills to the development of mathematics skills (dotted line). Entries with a double line represent skills shared between mathematics and reading. It is of course schematic, but we hope it highlights the clear differences between the types of skill required, together with the clear overlap in the need for executive function skills.

## Discussion

In the introduction, we posed three questions: Why is there comorbidity, why is there differentiation, and what are the implications for subsequent progress? We proposed that the process of 360DA could cast light on these issues and are now in position to take stock. We start with the four perspectives then return to the three questions.

### Theoretical Perspective

In terms of theoretical interpretation, the results go beyond all of the extant theories. The phonological deficit hypothesis is best seen as at the symptom level rather than a causal explanation. It is unable to account for the dissociation between mathematics and reading and excludes non-language components of reading such as eye movements. The executive function/attention hypotheses may be seen as reflecting symptoms of some underlying unspecified cause but again do not differentiate between MD and RD. Magnocellular deficit provides a systematic explanation for difficulties in reading but provides no explanation for the comorbidity between the two. Our multi-level explanation in terms of automatization deficit, cerebellar deficit, and procedural learning deficit does provide a principled explanation of the range of problems in reading disability but was not designed to provide an explanation for mathematics disability. None of these theories explicitly takes into account the affective dimension, and therefore, in our view all these theories should be augmented to create a two stage developmental explanation with first a developmental delay leading to lack of school readiness for executive function, reading readiness, or math readiness as appropriate, which leads to school failures, for which the ensuing shame and stress lead to affective trauma and essentially disables learning in that context.

### Developmental Perspective

We defer analyses of the specific skills to the section on pedagogical perspective, and here, we focus on the generic cognitive development processes that take place in pre-school and early-school years. Piaget’s well-established stages of cognitive development do provide some pointers, with the pre-operational stage and concrete operations stage of particular relevance. Furthermore, as outlined earlier, Piaget’s key concepts of internalization and bricolage are particularly relevant. Internalization (which is similar to automatization) indicates that a concept or skill has become so well assimilated that it can actually be used in more complex operations, the process he referred to as bricolage. Letter-sound pairings and number bonds would be examples for reading and arithmetic, respectively.

The more recent developmental frameworks tend to emphasize the issue of executive function and its development, as discussed earlier. The need for executive function skills to have developed sufficiently to support classroom readiness, working memory, and processing speed is of course critical to the transition to formal schooling. The evidence that SES disadvantage leads to delay in executive function development (Waters et al., 2021) is particularly concerning in this regard.

In terms of the generic developmental perspective, we would suggest that the issues involved are equivalent for both RD and MD and therefore contribute to the shared (comorbidity) data.

### Affective Perspective

The affective perspective is particularly interesting, potentially contributing either to increased differentiation or increased comorbidity, depending on the degree to which stress and anxiety transfer from the weakest skill to other areas of schoolwork. As noted earlier, situation-specific stress leads to marked impairment in the ability to learn in that situation (reading in the case of RD). However, impaired performance on reading might well lead not only to reading-related stress but also to the more general (and equally toxic) feeling of shame, and shame will attach to the more general school environment. Shame has similar consequences in terms of blocking as does stress (Cesare et al., 2018) and will therefore impair not only reading but also mathematics.

In either case, the affective dimension is a potent blocker of learning, but very much more pervasive if allowed to generalize. Consequently, it is very important for a teacher to clearly differentiate between the two disciplines, so that the better subject is not dragged down by the weaker one. Indeed, it may well be that an early specific diagnosis of LD (or MD) will serve to avoid this cross-subject transfer of shame.

### Pedagogical Perspective

In terms of pedagogic interpretation, a key implication is that for children at risk of mathematics failure, spatial, and conceptual play will be particularly important for scaffolding the necessary underlying skills. Computer games can provide invaluable judgment-free support (Rasanen et al., 2009). For children at risk of reading failure, eye fixation ability perhaps *via* non-reading games (Franceschini et al., 2013) will be an invaluable adjunct to phonological support.

As noted above, a lack of mathematics readiness or reading readiness or classroom readiness at the start of formal instruction is the trigger for the school failure that leads to further problems. It may well be that delaying the start of formal reading instruction or formal math instruction until executive function is well developed as practiced in the Scandinavian countries, would be beneficial for all children. Interestingly, work by Suggate et al. (2013) in New Zealand showed that delaying the start of formal reading instruction by a year led to no noticeable problems at the age of 10 for high performing children and much better performance for low performing children at that age.

### Why Differentiation?

Task analysis indicated that reading has a much stronger reliance on language skills, on eye fixation ability, and on visual/verbal co-processing, skills which may be characterized as procedural, relating to a cerebellar/cortical circuitry. By contrast, the specific requirements for early mathematics are more conceptual, and are scaffolded by spatial abilities, thereby implicating the hippocampal circuitry.

It may well be that the gender dissociation found for the specific forms of RD and MD may also be attributable to differences in these underlying skills. For example, a child with specific reading difficulties may have specific problems in phonological skills or a specific difficulty in steadily fixating the material on the page, both of which will lead to impaired reading ability. By contrast, a child with problems in understanding the number line or its spatial representation will have conceptual difficulties leading to impairments in mathematical learning.

This explanation is consistent with long-standing evidence that girls tend to have a relative advantage in language skills, whereas boys have a relative advantage with spatial skills, though there is a strong movement to challenge these beliefs (Hyde, 2005). Recent large-scale evidence does suggest that there is a significant advantage for boys in spatial processing (Wong and Yeung, 2019) and in spatial processing of numbers (Zhang et al., 2020). The importance of spatial processing for early mathematics was confirmed by a meta-analysis of 75 studies (Peng et al., 2020) that established key attributes as deficits in phonological processing, processing speed, working memory, attention, executive function, and visuospatial skills. Gender differences in language have been confirmed by large-scale studies in Germany (Lange et al., 2016) and Taiwan (Lung et al., 2011), and a very large recent US analysis (Reilly et al., 2019) concluded that “*language and verbal abilities represent one exception to the rule of gender similarities.*”

Interestingly, this phonological/spatial dissociation hypothesis is directly consistent with the Moll et al. (2014). German study cited earlier which established that there was greater comorbidity between SD and MD than between SD and RD. Since both reading and spelling are dependent on phonological processing, this dissociation between them highlights the importance of the dissociating sub-skill, namely binocular fixation.

In conclusion, the 360DA framework has revealed both commonalities and dissociations between MD and RD. The comorbidities may be attributed to two common aspects, initially a developmental delay in some of the underlying skills lacking scaffolding from visual and language skills, respectively, followed by the trauma and learning disability attributable to failure to acquire these school attributes.

### Why Comorbidity?

As demonstrated above, there is scope for comorbidity in three of the four perspectives. First, there are shared needs for fact internalization in terms of the letter facts and number facts, and even though the internalization process is driven largely by the procedural system, the initial stages involve the two systems working together (Doyon et al., 2003). Second, in terms of the developmental perspective, a delay in developing the executive function skills needed to cope with classroom instruction will—irrespective of reading readiness or arithmetic readiness – lead to problems in carrying out the teacher’s instructions, and hence failure at both reading and arithmetic. Early failures of this type will lead to stress, and probably shame, thereby triggering the potent affect dimension, effectively disabling the learning process for the skill involved. If the stress is limited to just arithmetic or just reading, this will lead to (specific) MD or RD, respectively, whereas if it generalizes to the entire school situation, the disability will be comorbid for MD and RD, and the child will probably show clear attention deficits as a consequence of the “fight, flight, or freeze” responses resulting from the HPA axis cortisol deposits.

### How Should We Proceed?

Arguably the most effective way forward is to acknowledge the crucial role of the classroom teacher in classroom learning and support. As we have stressed above, there is a major risk that an initial delay in executive function development leads to a deficit in classroom readiness, leading to failure, to situation specific stress, leading to a learning disability, which may or may not generalize to the broader classroom environment. By the time, a child is referred to an educational psychologist or other expert, the failures will have occurred and the stress patterns will have been set up. It should surely be an intrinsic component of teacher training to be fully aware of the toxic effects of failure stress on learning abilities. The 360DA process provides a simple framework for teacher empowerment in this process.

In terms of assessment, a key issue is “assessment for support.” Information processing analysis such as executive function, working memory, and speed of processing provides little explicit basis for support unless this leads to a clear problem, though see Diamond (2013) for advocacy of early support for executive function. Surprisingly, effective, hierarchical, task analysis of the skills involved has not been the target of systematic approaches within educational settings. By contrast, task analysis was at one stage the major component of pedagogical support at the individual level (Gagne, 1965).

We advocate the use of the 360DA process outlined here, aimed at developing an individually based intervention program date designed around the theoretical cognitive, affective, and pedagogical approach.

## Conclusion

Fifty years ago, the theoretical approach to the developmental disorders was in terms of minimal brain dysfunction (Wender, 1978), and support methods included both occupational therapy and specific task-focused approaches (Gagne, 1965), and in the United States the opportunity to repeat a year, thereby allowing developmental maturation to take place.

Moving forward, phonological deficit theorists take the commonality between RD and MD to advocate that language disorder may be at the root of both (Snowling et al., 2021). By contrast, advocates of procedural learning deficits take the commonality as indicating further support for procedural deficits in both disorders (Evans and Ullman, 2016). From our own perspective, in this article, we highlight the importance of our automatization deficit framework, together with our procedural learning deficit framework. To take a devil’s advocate approach, it does appear these interpretations of the data reflect more the preconceptions of the theorists than an objective analysis of the way forward. An example, perhaps, of Maslow’s Hammer (*I suppose it is tempting, if the only tool you have is a hammer, to treat everything as if it were a nail*; Maslow, 1966).

We have, however, outlined a methodology for escaping from this scientific solipsism. First, incidence analysis does provide a clear theoretical dissociation at the group level highlighting the difference in incidence between male and female for both disorders. Second, task analysis highlights the multiple factors that can contribute to failure to acquire fluency in either skill. It also provides an analysis of the conceptual and applied scaffolding techniques that are used either naturally or *via* intervention to assist an individual child gaining necessary mastery. Third, the theoretical and task analyses do allow us to develop a principled analysis of the developmental task requirements for each domain, thereby allowing us to pinpoint not only areas, where each individual may be affected, but, ideally, interventions designed specifically to address each limitation.

In summary, MD and RD overlap in some areas and are distinct in other areas. Starting with the later developmental stages, irrespective of the causes of the failure, failure at either mathematics or reading will lead to trauma and stress. This essentially disables the learning process for that domain, leading to the development of a toxic, learned helplessness stage, where a child is not able to focus effectively on the task in hand.

Second, it is likely that the rote learning aspect of the conversion of the symbols on the page to their mental concepts is common to both disorders. Furthermore, there are overlaps between learning the grapheme-phoneme conversion rules, and the rules of number, but in fact the rules of number are generally learned by a rote repetition strategy, thereby leading to problems from knowledge internalization, whereas the grapheme-phoneme conversion problem is very much more complex requiring high levels of phonemic awareness and tolerance of arbitrary and inconsistent mappings.

In conclusion, we developed the 360DA framework to provide a methodology for “due diligence” in the developmental disorders, attempting to avoid premature commitment to a single perspective, or analysis of a single disorder. We applied the two-stage methodology of 360DA, first presenting the evidence on four perspectives at the group level – theory, development, affect, pedagogy – and then integrating these findings at the explanatory, theoretical, and pedagogical levels. This comprehensive and systematic approach led to significant integration, with clear implications for the major aims of research in this area – to enhance the education of each individual child, and to improve the overall educational system. The analyses are incomplete, but novel, and we hope they provide the methodology and the inspiration for further researchers to break out of the straitjacket of discipline-limited approaches.

## Author Contributions

RN and AF contributed to the conceptual analysis and wrote the manuscript. All authors contributed to the article and approved the submitted version

## Funding

This study was funded by the Edgehill University. AF is an Emeritus professor currently unfunded.

## Conflict of Interest

The authors declare that the research was conducted in the absence of any commercial or financial relationships that could be construed as a potential conflict of interest.

## Publisher’s Note

All claims expressed in this article are solely those of the authors and do not necessarily represent those of their affiliated organizations, or those of the publisher, the editors and the reviewers. Any product that may be evaluated in this article, or claim that may be made by its manufacturer, is not guaranteed or endorsed by the publisher.

## Footnotes

^1^It is worth noting that they use the term procedural learning to refer to proceduralization of skill, the first stage in skill automatization (Anderson, 1982). By contrast, some theorists refer to the “serial reaction time task,” where a participant becomes faster when responding to a long repeating sequence of key presses, which is more appropriately labeled as “sequence-based statistical learning.” For true procedural learning, the participant needs to be consciously aware of the task, and to be able to consolidate the skills at least overnight.
